# Supplementary material for: Interpolation of microbiome composition in longitudinal data sets
Source: mBio. 2024 Aug 20;15(9):e01150-24. doi: 10.1128/mbio.01150-24 (PMC11389371; doi:10.1128/mbio.01150-24)
Supplement: Supplemental Material — Figures S1-S4; Tables S1 and S2. [file mbio.01150-24-s0001.pdf]

Supplemental Material

Interpolation of Microbiome Composition in Longitudinal Datasets

Omri Peleg and Elhanan Borenstein

Supplementary Figures

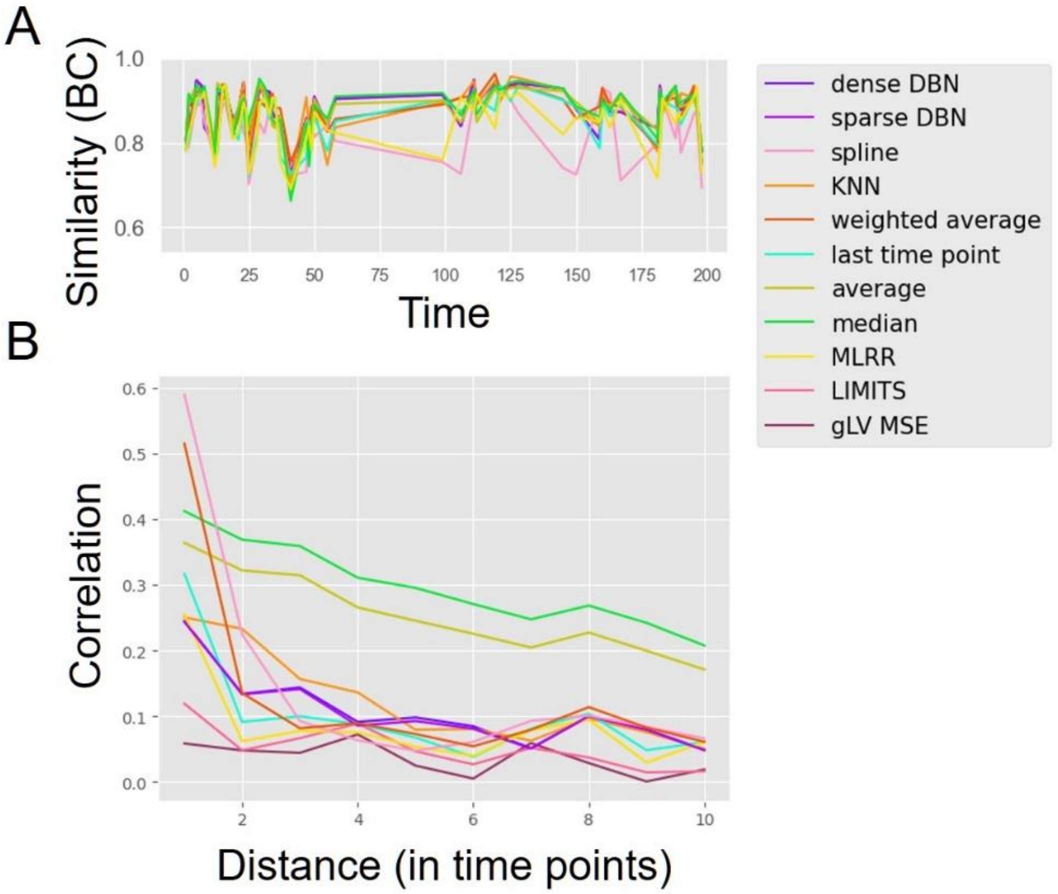

Figure S1: Interpolation accuracy across time. (A) Temporal interpolation accuracy for one specific individual ("ae") from the Poyet *et al*. dataset. The lines represent the Bray-Curtis similarity between the interpolation results and the real abundance in each method. **(B)** The average auto-correlation over all individuals between interpolation accuracy of a sample and the interpolation accuracy of a preceding sample across varying time intervals.

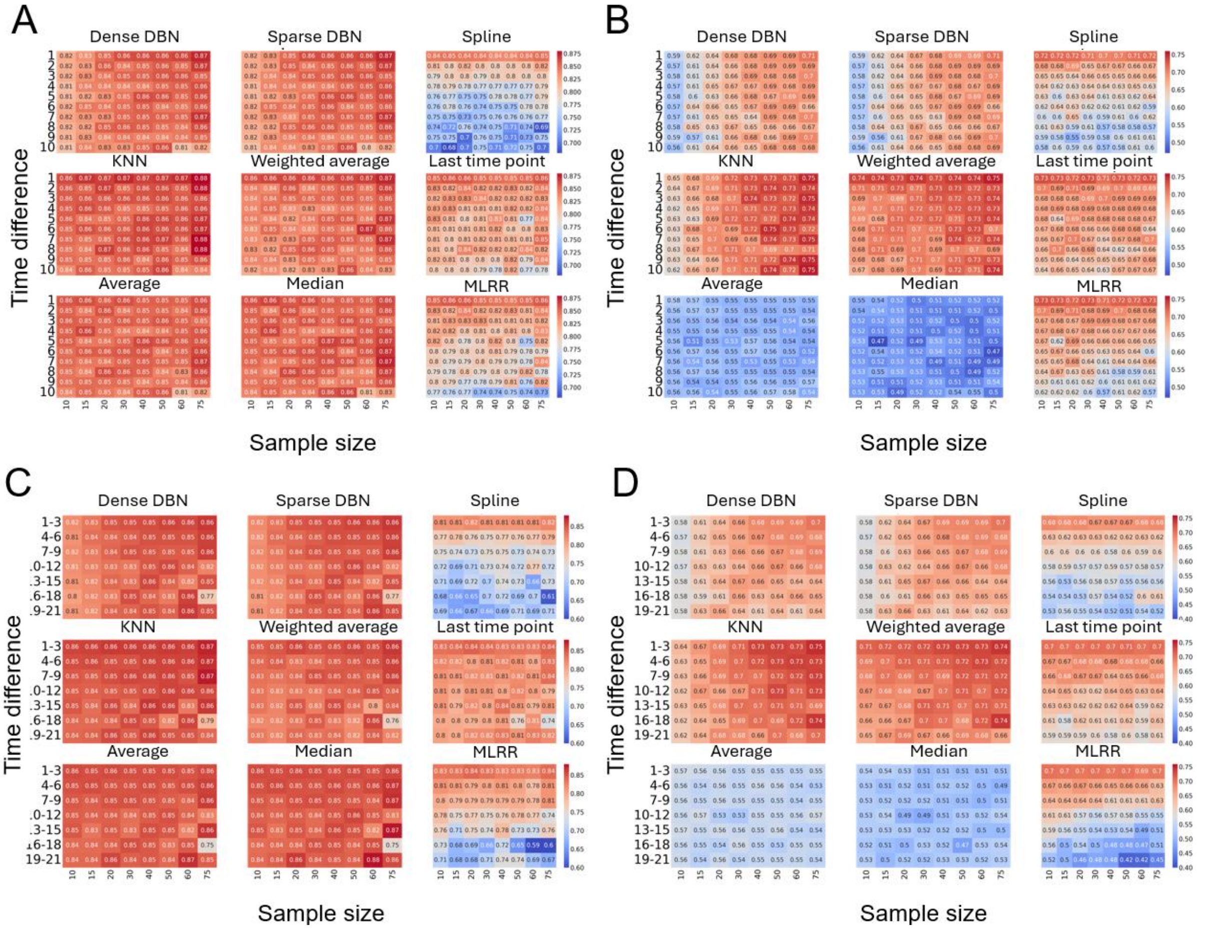

Figure S2: Interpolation accuracy is correlated with both sample size and the time interval between adjacent samples. Mean interpolation accuracy (across all individuals) for fixed sample sizes and time interval for **(A)** non-infants (Caporaso *et al*. and Poyet *et al.*) and **(B)** infants (Trosvik & Muinck), considering only sample size and time difference pairs that had at least 50 samples. Panels **(C)** and **(D)** expand panels **A** and **B**, respectively, reporting the impact of larger time intervals by grouping together a range of time intervals and reporting the mean interpolation accuracy for every range.

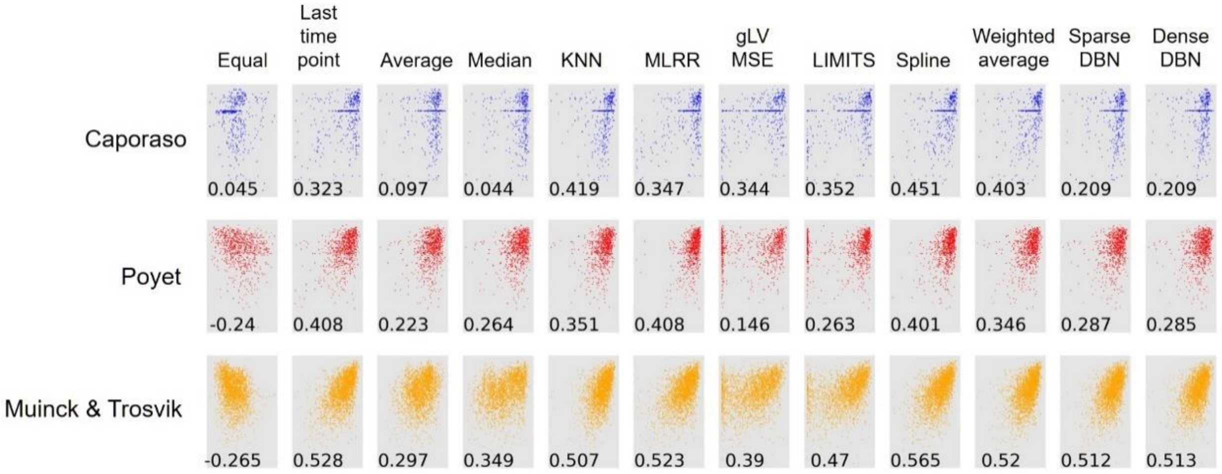

Figure S3: Interpolation accuracy and microbiome stability over time. Correlation between the interpolation accuracy at sample i and the stability of the microbiome around that sample (measured as the average BC distance between the samples at samples $i-2$ and $i-1$, $i-1$ and $i+1$, and $i+1$ and $i+2$). The Number at the bottom left of each panel denotes the spearman correlation.

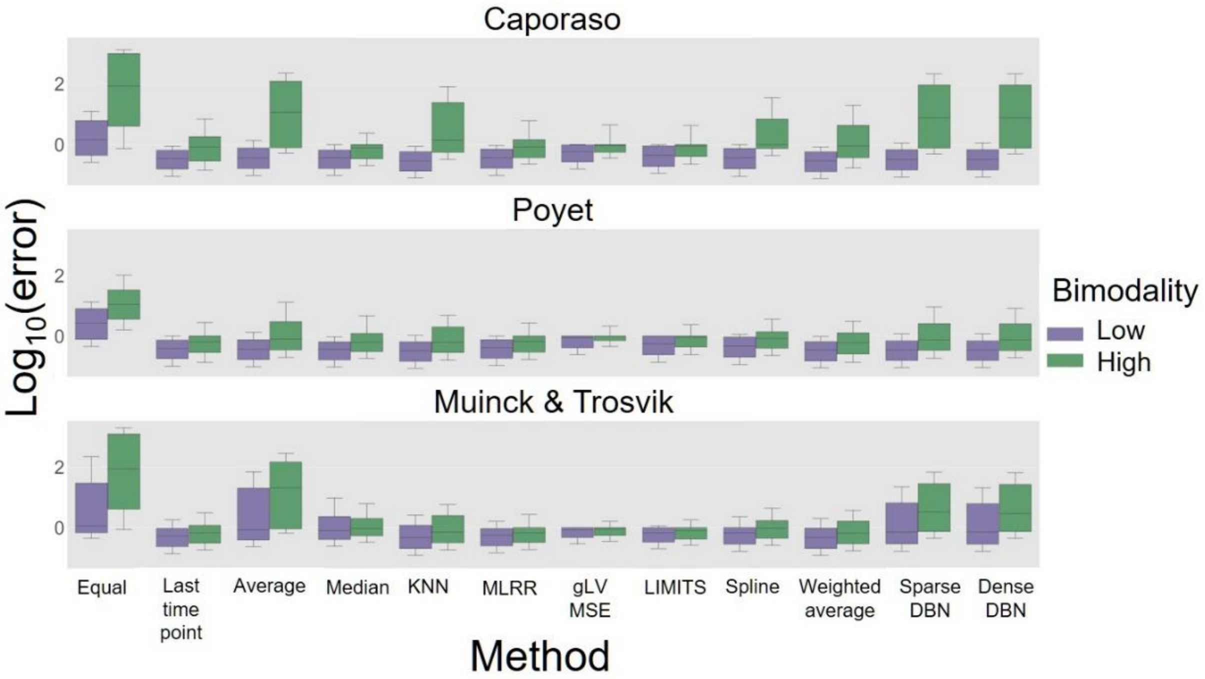

Figure S4: Taxa interpolation accuracy is linked to the stability of the taxa. Shown are boxplots denoting the distribution of relative error in taxa with low vs. high bimodality coefficient, for every taxon across all individuals and datasets. The cutoff for bimodality coefficient was set at 0.7, similarly to the 0.8 cutoff used in Gibbons *et al.*, 2017.

Supplementary Tables

	Equal	last time point	average	Median	KNN	MLRR	gLV MSE	LIMITS	spline	weighted average	sparse DBN	dense DBN
**Preceding time point interval infants**	-0.021	-0.047	-0.015	0.018	0.004	-0.121	-0.482	-0.421	-0.109	-0.004	-0.036	-0.031
**Preceding time point interval non-infants**	-0.024	-0.225	-0.089	-0.072	-0.144	-0.298	-0.717	-0.623	-0.308	-0.160	-0.158	-0.156
**Subsequent time point interval infants**	-0.027	-0.004	0.004	0.016	0.012	-0.012	-0.042	-0.033	-0.129	-0.021	0.005	0.007
**Subsequent time point interval non-infants**	-0.021	-0.018	-0.058	-0.040	-0.091	-0.016	-0.019	-0.020	-0.299	-0.130	-0.078	-0.080

Table S1: Spearman correlation between each method's interpolation accuracy and the time difference from the preceding time point and the subsequent time point. Results are divided to infants (Muinck & Trosvik) and non-infants (Caporaso *et al*. and Poyet *et al.*).

	Equal	last time point	average	Median	KNN	MLRR	gLV MSE	LIMITS	spline	weighted average	sparse DBN	dense DBN
**Preceding time point time difference**	0.3374	1E-09	0.9019	0.3658	0.0263	3E-84	9E-159	1E-219	1E-12	0.1892	4E-05	5E-05
**Preceding time point quality of interpolation**	9E-223	4E-134	5E-140	6E-172	3E-91	2E-98	9E-06	2E-11	0	0	3E-99	2E-98
**Samples count**	0.6787	0.9008	0.3592	0.1993	0.6111	0.2304	3E-06	0.4575	0.0766	0.5419	0.5232	0.6009
**Subsequent time point time difference**	0.3031	0.4037	0.4604	0.5001	0.2955	2.3E-06	0.0035	0.3597	1E-22	0.0019	0.5556	0.6349
**Subsequent time point quality of interpolation**	2E-222	2E-134	5E-135	4E-167	3E-90	2E-103	9E-07	3E-14	0	0	3E-100	1.8E-99
**Mean error**	0.0370	0.0868	0.0730	0.0743	0.0667	0.0984	0.2242	0.1816	0.0530	0.0541	0.0709	0.0712
**Error STD**	0.0328	0.0831	0.0654	0.0735	0.0689	0.0992	0.1381	0.1378	0.0559	0.0527	0.0722	0.0720

Table S2: P-value for each variable in every method using Wald test for LMM model, as well as the mean and standard deviation of the absolute error between the model's expected interpolation accuracy and the real interpolation accuracy across all time points.
